# Implementation of genomics in medical practice to deliver precision medicine for an Asian population

**DOI:** 10.1038/s41525-019-0085-8

**Published:** 2019-06-07

**Authors:** Yasmin Bylstra, Sonia Davila, Weng Khong Lim, Ryanne Wu, Jing Xian Teo, Sylvia Kam, Tamra Lysaght, Steve Rozen, Bin Tean Teh, Khung Keong Yeo, Stuart A. Cook, Patrick Tan, Saumya Shekhar Jamuar

**Affiliations:** 1 SingHealth Duke-NUS Institute of Precision Medicine, Singapore, Singapore; 2 Cardiovascular and Metabolic Disorders, Duke-NUS Medical School, Singapore, Singapore; 3 Cancer and Stem Biology, Duke-NUS Medical School, Singapore, Singapore; 4 Center for Applied Genomics and Precision Medicine, Department of Medicine, Duke University School of Medicine, Durham, NC USA; 5 Durham Veteran Affairs Cooperative Studies Program Epidemiology Center, Durham, NC USA; 6 Centre of Biomedical Ethics, National University Hospital, Singapore, Singapore; 7 National Cancer Centre, Singapore, Singapore; 8 Department of Cardiology, National Heart Centre, Singapore, Singapore; 9 Biomedical Research Council, Agency for Science, Technology and Research, Singapore, Singapore; 10 Department of Paediatrics, KK Women’s and Children’s Hospital, Singapore, Singapore; 11 Paediatric Academic Clinical Programme, Duke-NUS Medical School, Singapore, Singapore; 12 SingHealth Duke-NUS Genomic Medicine Center, Singapore, Singapore

**Keywords:** Genetics research, Genetic testing, Preventive medicine

## Abstract

Whilst the underlying principles of precision medicine are comparable across the globe, genomic references, health practices, costs and discrimination policies differ in Asian settings compared to the reported initiatives involving European-derived populations. We have addressed these variables by developing an evolving reference base of genomic and phenotypic data and a framework to return medically significant variants to consenting research participants applicable for the Asian context. Targeting 10,000 participants, over 2000 Singaporeans, with no known pre-existing health conditions, have consented to an extensive clinical health screen, family health history collection, genome sequencing and ongoing follow-up. Genomic variants in a subset of genes associated with Mendelian disorders and drug responses are analysed using an in-house bioinformatics pipeline. A multidisciplinary team reviews the classification of variants and a research report is generated. Medically significant variants are returned to consenting participants through a bespoke return-of-result genomics clinic. Variant validation and subsequent clinical referral are advised as appropriate. The design and implementation of this flexible learning framework enables a cohort of detailed phenotyping and genotyping of healthy Singaporeans to be established and the frequency of disease-causing variants in this population to be determined. Our findings will contribute to international precision medicine initiatives, bridging gaps with ethnic-specific data and insights from this understudied population.

## Introduction

The integration of genomic technologies is transforming healthcare by enabling targeted screening, diagnosis and treatment for disease management. There are now several emerging efforts demonstrating the application of genomics for medical care, in both healthy and disease cohorts, at both institutional and national levels.^1–5^ Many of these initiatives have involved the collation of large-scale data from populations of European ancestry incorporated into Western health practices. In the past few years, genomics has also been adopted for clinical care in the Asia Pacific region.^6^ However, optimal utility continues to be challenged by the lack of disease registries and large-scale genomic and clinical data sets specific to Asian ancestry.^6,7^ Lack of such ethnic-specific data renders challenges in developing best practice precision medicine initiatives for local populations that will enhance existing health services.

To address these gaps, the SingHealth Duke-NUS Institute of PRecISion Medicine (PRISM) was established in 2016 to advance precision medicine efforts with a focus on Asian-specific clinical and genomic data. To foster a learning health system, this collaborative endeavour brings together both research (from Duke-NUS Medical School) and clinical (from SingHealth) expertise with the focus on two key components: (1) health data assembly and analytics, and (2) knowledge transmission for genomics implementation in the clinic.

We previously reported the development of an evolving aggregated genomic data set of Southeast Asians known as the Singapore Exome Consortium (SEC).^8^ The SEC is derived from individuals from the multi-ethnic Singaporean population, comprising of Chinese, Malay, Indian and other minority Asian ethnic groups, who have been recruited as healthy controls for genomic-based research studies within Singapore. A subset of SEC genomic data was obtained from a research program called SPECTRA, a prospective cohort of consented volunteers without significant comorbidities who are extensively phenotyped and undergo whole genome sequencing, allowing for clinical and genomic correlations. These participants consent for follow-up using electronic medical records (EMR) for up to 20 years. As such, this cohort provides a comprehensive healthy control data set for disease phenotypic and genomic studies relevant to Asian populations. In addition to the donation of biospecimens and health data for analysis, SPECTRA participants can also consent to receive genomic findings relevant to their medical care. Taking this into consideration, we have devised a model to return research results, which can then be used for clinical care and is complementary to our current healthcare system.

**Fig. 1:**
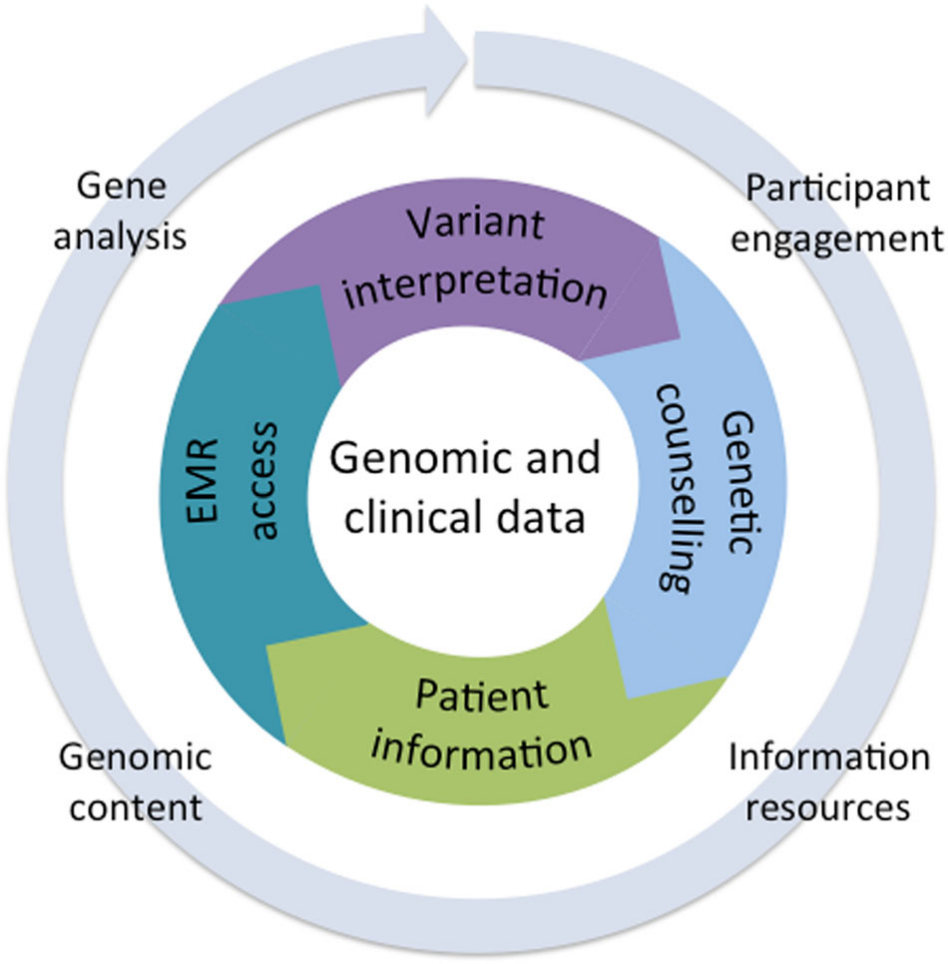
PRISM genomics implementation model. The inner circle represents the core components for delivery of the genomic data into medical practice. The outer circle represents variables that can be adapted as implementation progresses

Overall, the implementation of this study is aimed towards enabling PRISM to: (1) determine the range of Asian normality by detailed phenotyping and genotyping of healthy Singaporeans; (2) define the frequency of likely pathogenic and pathogenic variants within the Singaporean population and (3) establish a clinical framework to identify and return medically relevant genomic variants. This framework for delivery of research genomic variants has been formulated as a continual learning cycle where implementation can be monitored and modified over time with emerging evidence. The model contains core elements for data analysis and its transmission to participants with the provision of variable components to modify and enhance delivery over time, as demonstrated in Fig. 1. In this paper, we describe how the genomic and clinical data is collected, analysed and returned to participants so that research findings can be integrated with ongoing clinical care.

## Study design, participants and setting overview

**Fig. 2:**
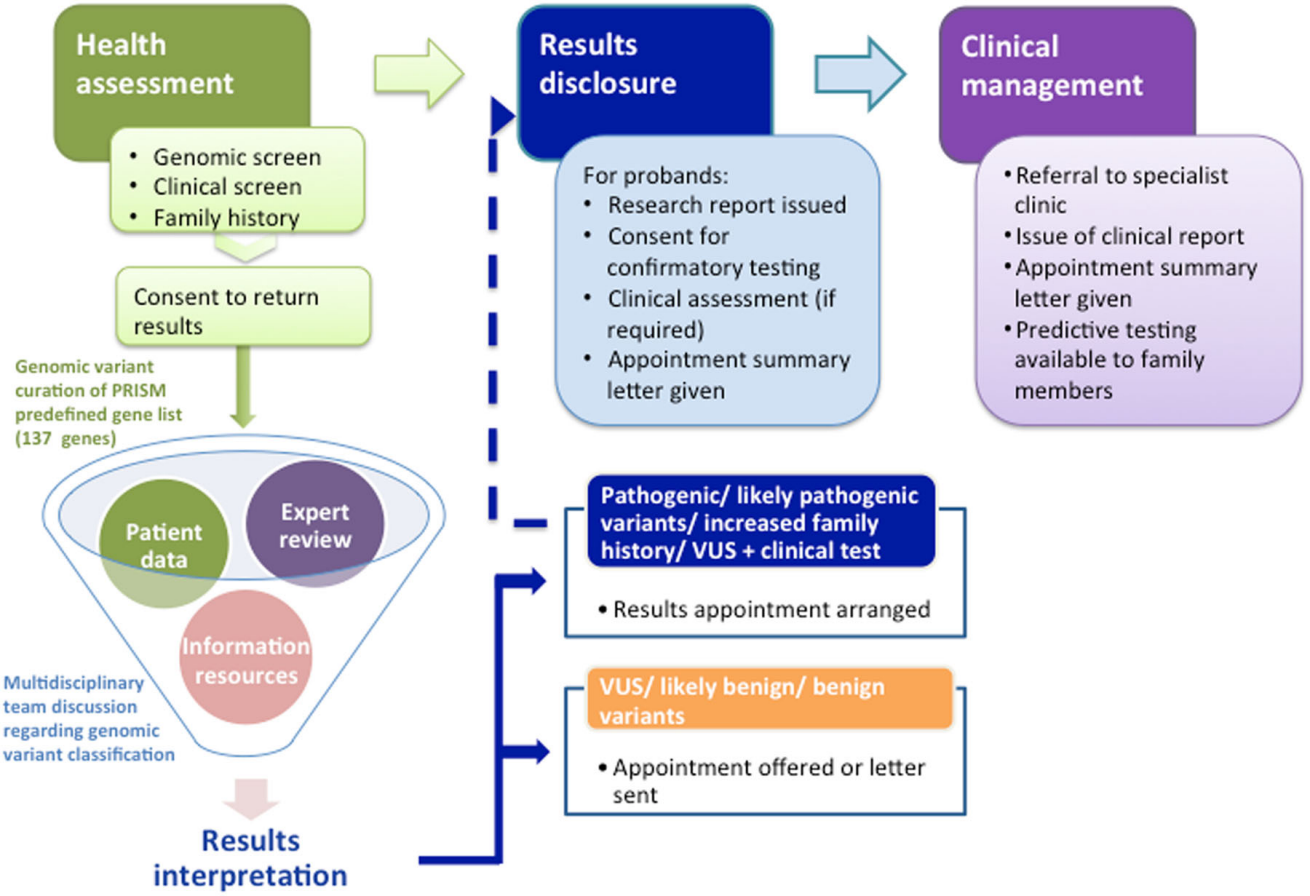
Flowchart representing an overview of participant engagement. Participant recruitment entails the collection of detailed phenotypic and genotypic information. Genomic variants are reviewed in a MDT meeting alongside associated clinical data. Those deemed medically relevant are returned to consenting participants through an onsite genomics clinic

The SPECTRA study is a prospective, longitudinal, observational study of the utility of genomics within routine medical care of Singaporeans (further details in Supplementary text). With an initial target of 10,000 volunteers, aged 18 years and above, to date over 2000 participants with no known pre-existing health conditions have consented to undergo a detailed health screen, collection of family health history (FHH) and lifestyle activity information and a genetic screen using whole genome sequencing technology. Ethics approval was obtained by the SingHealth Central Institutional Review Board in 2014. As such, the PRISM institute was purpose-built to include a research facility to correlate genomic and clinical data using bioinformatics analytics and a clinical facility comprising of genetic counselling, medical examination and clinical consultation rooms. This setting enables clinically relevant research findings to be communicated directly to the participants by the clinical genetics team. Participants found at increased risk because of a clinically validated genetic result or FHH are then referred to the appropriate specialist department within the hospital for ongoing management. Using existing hospital services for clinical screening, our team comprises of two clinical research coordinators, a laboratory assistant for sample preparation, two bioinformaticians, two genetic counsellors, a clinical geneticist and a board of directors to oversee the logistics and implementation. The overall process regarding recruitment to results analysis, disclosure and follow-up is displayed in Fig. 2.

### Consent for return of genomic screen results

Participants are informed that only genes associated with clinical actionability will be reviewed and could indicate:

1. a diagnosis of a genetic condition that may have been unknown
2. an increased risk of developing a genetic condition
3. detection of carrier status for a Mendelian disease
4. information about response to drug administration

Participants are informed that a genetic counsellor is available during the recruitment appointment to aid in the decision regarding the option to receive genomic findings. Participants are also notified that they can withdraw consent at any time. Participants under 21 years of age in Singapore are considered minors and therefore require additional parental or guardian consent.^9^ Unlike other Asian countries,^10^ in this experience, individuals over 21 years consent to participate autonomously and independently in the consent taking process without involvement from their family or community.

### Personal and Family Health Data collection

Self-reported baseline demographics, socioeconomic, lifestyle activity, medications and personal health history information are collected by questionnaire. FHH is obtained by an online web application, MeTree, which captures a three-generation pedigree.^11,12^ MeTree adopts family history collection based on a Western kinship system however family relationships can have different connotations in an Asian culture. For example, “aunty” and “uncle” are terms used to address elders in the community out of respect. To avoid any confusion, training and handouts were provided to the clinical research coordinators to assist the collection of relevant family history and it is explained to the participants that information from only blood-related family members is relevant.

### Gene panel

A gene list was devised by genetics experts from multiple disciplines within SingHealth to target analysis to genes that are documented to be clinically significant and relevant to the Singaporean population. The first version was developed in 2016 and contains the 59 recommended genes by American College of Medical Genetics and Genomics (ACMG),^13^ and an additional 60 genes. The additional genes considered for analysis are based on the reported experience of international genomics experts and the local experience of genetic conditions found prevalent in Southeast Asian populations. Additionally, our SEC local genomic database is analysed to understand carrier prevalence of genetic conditions that are more common in the local population, including diseases such as thalassaemia (1 in 25), citrin deficiency (1 in 41) and Wilson disease (1 in 103).^8,14–18^ The ACMG framework was used as a guide for assessing the clinical actionability of genes in our panel.^19^ Subsequently, the screening recommendations for each associated genetic condition were then reviewed to ensure that a management plan for ongoing follow-up could be offered in the Singapore context. As the type of consent regarding the return of genomic findings is broad, this enables flexibility to modify this gene list as data regarding relevance to this population emerges, currently on a two yearly basis.

Collectively, the PRISM gene list is associated with 77 Mendelian conditions, of which 88 genes are associated with autosomal dominant conditions, 26 genes with autosomal recessive conditions and 5 associated with X-linked conditions. In addition, 43 pharmacogenomic variants occurring in 23 genes with level 1A and 1B evidence of drug association according to PharmGKB are annotated (www.pharmgkb.org). The gene list has since been modified to include three further genes associated with familial hypercholesterolemia (*APOB, LDLRAP1, PCSK9*) and the gene *CYP21A2* associated with autosomal recessive inheritance congenital adrenal hyperplasia has been removed due to technical issues in accurately calling variants due to the presence of a pseudogene. Additionally, six genes (*KCNE3*, *CACNA1C*, *CACNB2*, *GPD1L*, *HCN4*, *SCN3B*) were removed from the PRISM gene list because the disease association with Brugada syndrome was classified as “Disputed” in ClinGen.^20^ In addition, there is limited evidence for the association of *HCN4* with familial thoracic aortic aneurysm and aortic dissection, and no reported evidence for the association of *CACNB2* with hypertrophic cardiomyopathy.^21^ The PRISM gene list version 2 contains 115 genes associated with Mendelian disorders and 22 pharmacogenomic genes (further details in Supplementary text).

### Genome sequencing and variant classification

Whole genome sequencing is performed on DNA extracted from peripheral blood using the Illumina platform. Bioinformatic analysis is performed as per best practice guidelines by the Genome Analysis Tool Kit (GATK) team (further details in Supplementary text). Variants reported as likely pathogenic or pathogenic by ClinVar or disease causing by HGMD in both intronic and exonic regions of genes in our gene list are selected for manual curation. In addition, as these mutation databases are known to be biased against non-European populations,^14,22^ we include novel loss of function variants (frameshift, nonsense, splice site, stop gain and stop loss) for manual curation. For each variant, the BAM file is visually inspected to confirm its validity. Of these variants, information in accordance with the ACMG variant classification guidelines^23^ is prepared and presented at a monthly multidisciplinary team (MDT) meeting comprising of clinical genetics, research and bioinformatics experts and critically reviewed.

### Multidisciplinary team meetings

To develop these meetings, invitations to the PRISM MDT variant classification meetings are sent to disease specialists within the hospital, with an interest or experience in inherited conditions. The variants for discussion are grouped according to their associated genetic condition and the relevant disease specialists are invited to attend. The data presented for the classification of variants is discussed until consensus regarding its pathogenicity is reached. In some cases supporting evidence is not sufficient to clearly classify the variant. For these variants, which are classified as variants of unknown significance (VUS), if additional information such as further FHH or a routine clinical laboratory test to ascertain the phenotype, such as a lipid profile, will assist with the interpretation, then these participants are also recontacted.

### Variant reclassification review

Although the clinical utility of variant reclassification with up-to-date information has been demonstrated,^20,24,25^ there are currently limited guidelines regarding the optimal timeframe for this to occur. The PRISM variant pipeline was developed in 2016 and then refined and updated in 2018. Until consensus regarding the frequency of variant classification is reached, the variants that border classification as VUS to likely pathogenic will be reviewed every 6–12 months. An update of the pipeline and variant annotation for all variants currently occurs on a two yearly basis. As the variants are manually curated, the updated list of annotated variants is incorporated into each run of the PRISM bioinformatics pipeline so that variants that have already been classified are easily identified. In addition, in the absence of local guidelines on variant classification, we have adopted and contextualised the ACMG variant classification, which is the current gold standard globally for this purpose.

## Return of genomic results

In order to return likely pathogenic and pathogenic results to participants, a PRISM research report is generated which was adapted from the MedSeq project.^26^ The report documents each monogenic condition and/or recessive carrier risk variant identified and contains brief information about the associated genetic condition, inheritance and health risks. Additionally, pharmacogenomic variants detailed with its associated drug and dosage recommendations are also listed. These recommendations are based on guidelines by the Dutch Pharmacogenetics Working Groups and PharmGKB, both accessible at www.pharmgkb.org. Genomic and participant particulars are re-identified for inclusion in the report. A research report is only generated for participants that have consented to receive genomic findings.

### Results disclosure

**Fig. 3:**
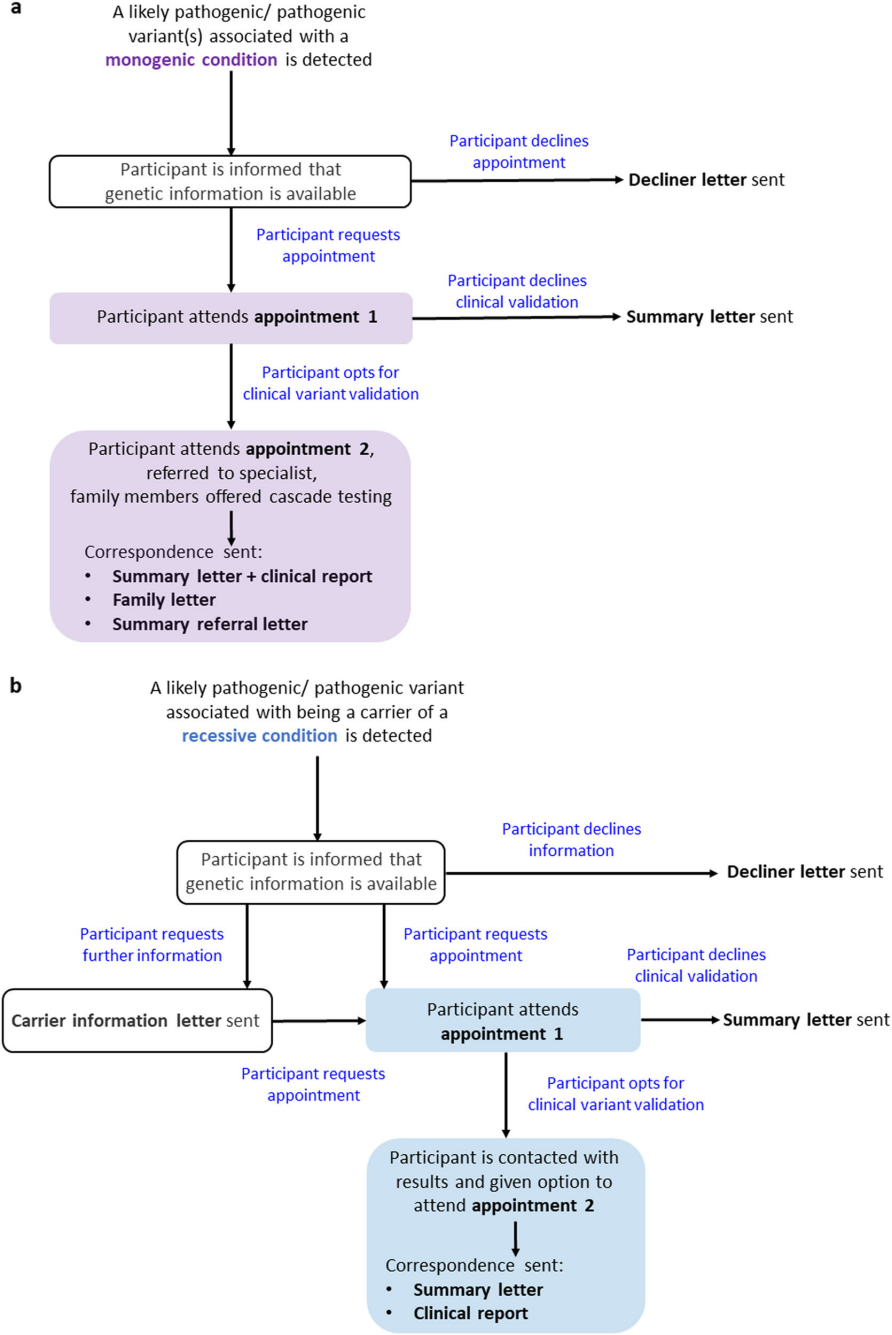
Flow diagram representing the process of recontacting participants that are either found to carry a likely pathogenic/ pathogenic variant(s) associated with developing a monogenic condition (**a**) or being a carrier of a recessive genetic condition (**b**)

For participants found to be at risk of developing a monogenic condition, a genetic counsellor contacts the participant by phone or email. A brief description of the study is provided and they are reminded that they have consented to receive genomic findings. Specific details regarding the variant or genetic condition are not disclosed over the phone. An appointment at the PRISM consultation room is then arranged to meet with the clinical genetics team. Initially, we also contacted participants identified to be a carrier of a recessive genetic condition and invited them to PRISM for an appointment. However, as the number of participants with a recessive carrier risk variant has increased significantly with little personal health implications, carrier participants are now contacted by phone and given the choice about whether to receive variant results by email, letter or in person (Fig. 3).

Participants who attend an appointment at the PRISM clinic meet with a genetic counsellor and clinical geneticist for an in-depth discussion about genomic sequencing, the list of genes that were analysed, variants detected, associated health risk information, inheritance, penetrance and implications for family members. If relevant to the variant detected, a physical examination may also be performed. The pharmacogenomic association variants are also reviewed and participants are given the option to download a mobile application (ggcME™) provided by Global Gene Corporation Pte. Ltd. (GGC), which enables them to keep track of the suitability of prescribed medications. With consent, PRISM provides GGC the pharmacogenomic data of these participants, so that they can enter a drug prescription and understand its suitability.

### Clinical Sanger validation

Participants who are either at risk of a monogenic condition or found to be a carrier of a recessive genetic condition are given the opportunity to have the variant clinically validated by Sanger sequencing at a certified laboratory. This requires reconsent and a new blood sample is collected for variant analysis. Currently, there is no charge to participants for clinical validation. The genetic counsellor explores the psychosocial impact of receiving such results, any possible genetic discrimination, intention of sharing this information with family members and their possible responses. These issues are revisited at each further contact. Participants are then invited back to the clinic to receive the clinical validation results with further discussion regarding medical screening specific to their age and health history. For those participants who opt out of validation, it is explained and subsequently highlighted in a consultation follow up letter, that the research results cannot be used for diagnostic or clinical management purposes. They are encouraged to contact PRISM to discuss validation at any stage in the future should they reconsider.

### Clinical follow-up

Participants identified at risk of a monogenic condition are referred to the appropriate specialist for ongoing care and their clinical validation report is submitted into their EMR. In addition, cascade testing is offered to their family members. Following their consultation at PRISM, each participant receives the PRISM research report and a summary of each consultation. This summary appointment letter states that participants may be contacted in the future if new genomic information becomes available. If relevant, they also receive the clinical validation report and an at-risk family letter for circulation amongst their family members describing the variant detected and its associated genetic condition, brief health implications for carriers and an invitation for family members to discuss further with PRISM.

### Family health history risk

At recruitment, a three-generation FHH is collected alongside genomic and phenotypic data. In some cases, FHH indicates an increased risk towards developing a genetic condition in absence of a genomic indication. Participants with a familial increased risk are also invited back to the clinic to discuss their FHH and screening recommendations based on their risk. They receive a letter summarizing the appointment and a referral to the appropriate healthcare specialist for further review.

### Participant follow-up

Each participant’s preference and reasoning to receive or decline the research findings and clinical validation, as well as contact attempts, are documented to monitor and refine the return of results delivery. Carriers at risk of a monogenic condition are also contacted by a PRISM genetic counsellor on a yearly basis for support and review around adaption and psychosocial impact of being a carrier, health behaviours and review of screening and communication of gene variants with family members. In addition, any future participant admission to SingHealth hospital for screening or diagnostic care can be tracked through the EMR. Information regarding hospital admissions and outpatient appointments, subsequent diagnosis, lab results and prescriptions is deposited directly to PRISM and updated on a 24-h basis. This enables adherence to recommended clinical follow-up based on genomic, clinical or FHH to be monitored and assessed for clinical impact.

## Discussion

In this report, we have outlined our strategy of integrating genomics into our local healthcare system, adopting well-established practices, such as genomic sequencing, bioinformatics analysis and reporting of the results, and integrating it with locally available resources and expertise. However, there are unique challenges and considerations when implementing this genomics framework in the Asian context, which are discussed below.

### Consent to return results

To expand the clinical application of this genomics research we designed a framework to return medically significant findings to the research participants. The consent process involving the return of results is two-staged: a broad research consent to return genomic variants and then a targeted clinical consent for variant validation. These are aspects that we have carefully considered during the design of this learning framework and will continue to modify as participant experiences become apparent. Unlike in the clinical context for reporting secondary findings,^13^ there are currently no guiding principles regarding the return of research results. Briefly, considerations which have been previously raised regarding the return of results include: benefit to research participants if the findings are not clinically valid or actionable,^27,28^ health care providers not being sufficiently trained for the disclosure^29^ and infrastructure requirements and costs.^30,31^

To alleviate some of these concerns, it has been suggested to advise participants of the disclosure policy at recruitment, restricting reportable variants to those of known clinically actionable implications, and provide participants the option to receive these results.^32^ In Singapore, there are no obligations for researchers to return research results or incidental findings to participants. Translational genomics research in Singapore is regulated by the 2015 Human Biomedical Research Act^33^ and states that the informed consent process should disclose whether incidental findings will be returned. Supporting this, an exploration of Singaporean patient preferences in the cancer, as well as paediatric rare disease settings, revealed that participants were in favour of being given the option to receive any clinically actionable germline results derived from research, and be made aware of this potential during the consent process.^34,35^ This current study will be able to provide further insights into the acceptability, preferences and utility of returning genomic research results to participants who are ostensibly healthy.

### Barriers to genetics services

In order for genomic results to be returned, we took into consideration some of the existing barriers to accessing genetics services that patients may currently experience. For example, in Singapore and its neighbouring countries, it is common that patients pay the full cost of genetic tests as there is no coverage from health insurance or government subsidy. Additionally, in the absence of genetic non-discrimination regulations for most Asian countries, genetic discrimination when taking insurance or employment is a possibility. Furthermore, social discrimination and stigmatisation can be experienced in association with a genetic risk and these sentiments have been echoed across Asia, for example, in Japan, Taiwan and Malaysia. Therefore, the cost of future surveillance and discrimination contributes to patients declining genetic testing in clinical settings.^10,36^

With these considerations in mind, PRISM devised a model that currently enables participants and their family members to access genetic testing, relevant to their medical care, with no out of pocket costs. They have the choice of whether to consent to clinical validation of the research findings with pre- and post-test genetic counselling discussions where issues of discrimination and stigmatization can be raised. This enables consideration of both the benefits and barriers of genetic testing before the results become integrated into their medical record where it might be reported to insurers or employers inadvertently or have implications socially.

### Health screening

The concept of health screening for prevention and early detection can have mixed acceptance and perceptions from Asian communities have been reported. One common viewpoint that has surfaced from population screening programmes in Asia is that the concern of an abnormality being detected outweighs any health benefits and this prevents uptake.^37,38^ Similarly, in absence of personal or family history, many participants in our study have no experiential knowledge of the condition for which they are at risk of developing. Given that we are returning results that are personalized and targeted to each participant with the addition of pre- and post-test counselling and provision of information resources, we can then monitor screening adherence in this cohort through EMR and patient follow-up.

### Clinician engagement

The application of this framework also relies on the engagement of the SingHealth medical specialists in fields such as cardiology, oncology and metabolism, amongst others. To assist with the variant classification process, we draw on their clinical expertise at our variant curation MDT meetings. This arrangement also creates the opportunity to raise awareness and educate regarding the clinical utility of genomics applications and the potential impact for patient management. It is acknowledged that despite the numerous applications of genomics in medical care, many healthcare professionals have not incorporated genomics into their practice. Reasons for this include a lack of understanding of the tests and the implications of the results for clinical guidance, insufficient time to receive adequate training and lack of practice guidelines. In addition, there are limited genetics experts available to provide training.^39,40^ Engaging medical specialists in identifying clinically significant results provides an opportunity for experiential learning and stewardship towards the integration of genomics into day-to-day medical practice. In addition, new inter-departmental working relationships are fostered which will promote the continuity of patient care when the research participants transition into a clinical setting.

### Clinical utility of data captured

We have developed an evolving reference base of genomic and clinical data to capture Southeast Asian ancestry. The ability to monitor long-term participant outcomes broadens the clinical scope of this data set to understand disease development and progression amongst Singaporeans. This will facilitate the tailoring of targeted screening, diagnostics and therapeutic interventions specific to this population. This data set will enable further insights into the identification of genetic carriers to define disease prevalence, genotype-phenotype correlations, observation of subclinical phenotypes after medical and genomic screening, development of a curated genomic variant database for variant analysis and pharmacogenomic variant analysis and adverse drug association.

### Conclusion

With the ability to correlate genomic, phenotypic and FHH information and monitor participant health outcomes, our aim is to progressively embed genomics into mainstream healthcare practices. For translation into practice, we have adopted a flexible learning framework to allow for adaptation to unanticipated challenges faced in real life clinical settings. In addition, our implementation strategies can be monitored and adapted over time. Our experience and findings will also contribute to current research initiatives internationally, bridging gaps with ethnic-specific data from this understudied population.

### Reporting summary

Further information on research design is available in the Nature Research Reporting Summary linked to this article.

## Supplementary information

Supplementary Material

Reporting Summary

## Supplementary information

**Supplementary Information** accompanies the paper on the *npj Genomic Medicine* website (10.1038/s41525-019-0085-8).
