# Supplementary material for: Implementation of genomics in medical practice to deliver precision medicine for an Asian population
Source: NPJ Genom Med. 2019 Jun 7;4:12. doi: 10.1038/s41525-019-0085-8 (PMC6555782; doi:10.1038/s41525-019-0085-8)
Supplement: Supplementary file 1 — Supplementary Material [file 41525_2019_85_MOESM1_ESM.pdf]

Supplementary Methods

Recruitment

An advertisement in a local daily newspaper, The Straits Times (which has the largest viewership in Singapore), was placed in 2014 and 2018 for healthy volunteers to be involved in a Biobank Programme at the National Heart Centre Singapore. Participation was entirely voluntary without any financial compensation, except for reimbursement for transport related costs. Briefly, participants over the age of 18 years with no known pre-existing health conditions are eligible. A dedicated clinical research co-ordinator (CRC) ascertains eligibility of the volunteers over the phone and then books an appointment for further discussion around study participation.

Consent for participation

The scope of the consent permits demographic, personal and FHH to be collected as well as medical records to be accessed. Additionally, participants elect to opt in or out of the following: 1. Receipt of any abnormal findings detected through the health screen, 2. A follow-up by phone every 5 years for 10 years to review health status and 3. receipt of genomic findings with important health implications.

Phenotyping of participants in the SPECTRA cohort

The health screen associated with Biobank involves an ECG, office blood pressure, heart rate, height, weight and body impedance measurements. In addition, a cardiac magnetic resonance imaging (MRI) and blood test by venipuncture is performed. DNA is extracted from the blood donation for genomic sequencing and serum is stored for future use. Participants who have any abnormality detected through the health screen which requires medical follow-up, are excluded from genomic sequencing analysis.

After the study visit for Biobank, participants are later recontacted and invited to participate in an additional substudy called SingHEART (https://clinicaltrials.gov/ct2/show/study/NCT02791152). Collectively the Biobank and SingHEART programs are referred to as SPECTRA. The SingHEART study includes further detailed phenotyping comprising of 24 hour ambulatory blood pressure monitoring, coronary calcium scoring, ECG, activity and sleep tracker by a wearable, a full blood count, liver function test, renal panel and a fasting blood test to measure cholesterol and glucose. Further details regarding the SingHEART study protocol are described in Yap *et al*, 2019 (under

review). All data is stored in a de-identified research database on a physically isolated network with personal identifiers stored separately on hospital systems not accessible to researchers.

Genome sequencing

DNA was extracted from peripheral blood. Genome sequencing was then performed on de-identified DNA samples by third-party sequencing providers using the Illumina HiSeq X platform using standard protocols. Sequencing data was returned in the form of FASTQ files.

Bioinformatic analysis

Raw genomic sequencing data received from the sequencing providers were analyzed using an in-house high-performance computing (HPC) cluster according to best practices recommended by the Genome Analysis Tool Kit (GATK) team. Briefly, FASTQ files were aligned to the human reference genome (hs37d5) using BWA-MEM (version 0.7.1(19). The aligned reads were sorted and de-duplicated using SAMBLASTER (version 0.1.24)(20). We then used GATK version 3.8(21) to perform base quality score recalibration (BQSR). The HaplotypeCaller tool was used to generate intermediate genomic variant call files (gVCFs). Next, gVCF files from the entire cohort were jointly genotyped using the GenotypeGVCF tool. The cohort-level variant call file (VCF) was then processed using the variant quality score recalibration (VQSR) tool to further identify high-confidence SNPs and INDELs. The resulting variant call set was then annotated using the ANNOVAR (2016-02-01)(22) tool to incorporate information on gene, genetic change, protein change, type of mutation (frameshift, nonsense, nonsynonymous, splicing, and synonymous); prediction of the variant effect from multiple algorithms, population allele frequencies in external databases (e.g. GNomAD, TCGA, etc.), and our in-house SEC database of local variants.

PRISM gene list

Genes associated with monogenic conditions:

ABCD1 | ACTA2 | ACTC1 | ACVRL1 | APC | APOB | ATP7B | BCHE | BLM | BMPR1A | BRCA1 | BRCA2 | CACNA1C | CACNA1S | CACNB2 | CASQ2 | CDC73 | CDH1 | CFTR | CNBP | COL3A1 | COQ2 | COQ9 | CPT2 | DMD | DMPK | DSC2 | DSG2 | DSP | EMD | ENG | EPCAM | F5 | FBN1 | FH | FLCN | GAA | GCH1 | GLA | GPD1L | HAMP | HBA | HBB | HCN4 | HFE | HFE2 | HMBS | IDUA | KCNE1 | KCNE2 | KCNE3 | KCNH2 | KCNJ2 | KCNQ1 | KIT | LDLR | LDLRAP1 | LMNA | MEN1 MET | MLH1 | MLH3 | MSH2 | MSH6 | MUTYH | MYBPC3 | MYH11 | MYH7 | MYL2 | MYL3 | MYLK | NF2 | OTC | PAH PCBD1 | PCSK9 | PDGFRA | PKP2 | PLN | PMS2 | PRKAG2 | PRKAR1A | PROC | PROS1 | PTCH1 | PTEN | PTS | QDPR | RBM20 | RET | RYR1 | RYR2 | SCN1B | SCN3B | SCN5A | SDHAF2 | SDHB | SDHC | SDHD | SERPINA1 | SERPINC1 | SGCD | SLC25A13 | SLC37A4 | SLC7A9 | SMAD3 | SMAD4 | SMARCB1 | STK11 | TGFB3 | TGFBR1 | TGFBR2 | TMEM43 | TNNI3 | TNNT2 | TP53 |

TPM1 | TSC1 | TSC2 | VHL

Genes with pharmacogenomic associations:
ANKK1 (PA134872551) | CFTR (PA109) | CYP2B6 (PA123) | CYP2C19 (PA124) | CYP2C9 (PA126) | CYP2D6 (PA128) | CYP3A5 (PA131) | CYP4F2 (PA27121) | DPYD (PA145) | EGFR (PA7360) | G6PD (PA28469) | HLA-B*15:02:01 | HLA-B*57:01:01 | HLA-B*58:01 | IFNL3 (PA134952671) | IFNL3 (PA134952671) | IFNL4 (PA166049147) | MT-RNR1 (PA31274) | NUDT15 (PA134963132) SLCO1B1 (PA134865839) | UGT1A1 (PA420) | VKORC1 (PA133787052) | XPC (PA37413)
